# Exploring the Therapeutic Potential of Essential Oils of the Valdivian Rainforest (*Drimys winteri* and *Laureliopsis philippiana*) for Sustainable Udder Health in Dairy Systems

**DOI:** 10.3390/ani16030445

**Published:** 2026-02-01

**Authors:** Isavo Vera, Leslie Vera, Diego Cabrapán, Paola Ramos, Fernando Ulloa, Diana Pantoja, Florencia Aranguiz, Martina Jacobs, Nicole Rojas, María Daniella Carretta, Flavia Bruna, Jessica Bravo, Javiera Bahamonde

**Affiliations:** 1 Instituto de Farmacología y Morfofisiología, Facultad de Ciencias Veterinarias, Universidad Austral de Chile, Valdivia 5090000, Chile; isavo.vera@alumnos.uach.cl (I.V.); leslie.vera@alumnos.uach.cl (L.V.); diego.cabrapan@alumnos.uach.cl (D.C.); diana.pantoja@uach.cl (D.P.); florencia.aranguiz@alumnos.uach.cl (F.A.); dcarreta@uach.cl (M.D.C.); 2 Laboratorio de Productos Naturales Bioactivos, Centro de Investigación Biomédica, Facultad de Medicina, Universidad Diego Portales, Santiago 8370007, Chile; proyectos.pramos@gmail.com (P.R.); martinajacobs@ug.uchile.cl (M.J.); nicole.rojas.lza@gmail.com (N.R.); jessica.bravo@mail.udp.cl (J.B.); 3 Instituto de Medicina Preventiva Veterinaria, Facultad de Ciencias Veterinarias, Universidad Austral de Chile, Valdivia 5090000, Chile; fernando.ulloa@uach.cl; 4 Escuela de Graduados, Facultad de Ciencias Veterinarias, Universidad Austral de Chile, Valdivia 5090000, Chile; 5 Laboratorio de Hormonas y Biología del Cáncer, Instituto de Medicina y Biología Experimental de Cuyo (IMBECU), CONICET CCT-Mendoza UNCuyo, Mendoza CP 5500, Argentina; flabruna@gmail.com; 6 Centro de Investigaciones Odontológicas, Facultad de Odontología, UNCuyo, Mendoza CP 5500, Argentina

**Keywords:** bovine mastitis, *Laureliopsis philippiana*, *Drimys winteri*, essential oils (EOs), antimicrobial resistance (AMR), Valdivian temperate rainforest, phytotherapy, sustainable veterinary medicine, udder health, mycotic mastitis, *Pichia kudriavzevii*, *Staphylococcus aureus*, *Streptococcus uberis*

## Abstract

Bovine mastitis is a major bioeconomic and animal health challenge in dairy systems and is traditionally managed with intensive antibiotic therapy, contributing to antimicrobial resistance (AMR). This study explored the therapeutic potential of essential oils (EOs) from two native species of the Valdivian temperate rainforest, *Laureliopsis philippiana* (Tepa; LP_EO) and *Drimys winteri* (Canelo; DW_EO), against priority mastitis pathogens. Gas Chromatography–Mass Spectrometry (GC–MS) was used to characterize EO composition, and in vitro antibacterial and antifungal activities were evaluated against clinical isolates of *Staphylococcus aureus*, *Streptococcus uberis* and the azole-resistant yeast *Pichia kudriavzevii* by disk diffusion and broth microdilution assays. Both EOs were dominated by monoterpenes; LP_EO was richer in oxygenated monoterpenes (eucalyptol, terpinen-4-ol), whereas DW_EO showed a pinene-rich profile (β-pinene, α-pinene). DW_EO produced significantly larger inhibition zones than LP_EO against *S. aureus* and *P. kudriavzevii* and exhibited lower MIC_50_/MIC_90_ values for *S. aureus*, *S. uberis* and *P. kudriavzevii*. Notably, DW_EO showed a higher inhibitory activity against *P. kudriavzevii* with a MIC_90_ of 4 mg/mL. These findings support DW_EO as a high-potential dual-action phytotherapeutic candidate for developing formulations and complementary tools within sustainable bovine udder health and antimicrobial stewardship frameworks.

## Simple Summary

Bovine mastitis is a major health and economic constraint in dairy production and is commonly managed with antibiotics. However, intensive use of these drugs favors antimicrobial resistance, therapeutic failures and residues in milk, challenging One Health goals. In southern Chile, pasture-based herds are affected by bacterial mastitis mainly caused by *Staphylococcus aureus* and *Streptococcus uberis*, and, although far less common, also by fungal mastitis due to the azole-resistant yeast *Pichia kudriavzevii*. In this study, we investigated whether essential oils from two native Valdivian trees, *Drimys winteri* (Canelo) and *Laureliopsis philippiana* (Tepa), could inhibit these mastitis pathogens in the laboratory. We characterized their chemical composition and evaluated antibacterial and antifungal activity against clinical isolates from dairy cows with mastitis and type strains. Both essential oils showed inhibitory effects on *S. aureus* and *S. uberis*, but Canelo oil, rich in α-pinene and β-pinene, was consistently more active than Tepa. Notably, Canelo oil also exhibited clear inhibitory effects against *P. kudriavzevii*. Overall, our findings identify Canelo essential oil as a promising natural phytotherapeutic candidate for bovine mastitis, particularly for difficult-to-treat yeast mastitis, and justify further preclinical evaluation of formulations as adjuncts to conventional antimicrobial therapies in pasture-based dairy systems.

## 1. Introduction

Bovine mastitis remains one of the most prevalent and economically significant diseases affecting the dairy industry, representing a challenge that bridges animal welfare, food safety and sustainability. It is estimated that more than 70% of the economic losses associated with dairy cattle diseases are attributable to mastitis, mainly due to reduced milk yield, veterinary expenses, premature culling and decreased milk quality [1]. Within the One Health framework, mastitis is one of the primary causes of antimicrobial use in livestock, making it a key target for antimicrobial resistance (AMR) mitigation strategies [2,3].

Bovine mastitis is an inflammatory disease of the mammary gland initiated by the entry of microorganisms through the teat canal and activation of the innate immune system [4,5]. Pathogen-associated molecular patterns are detected by mammary epithelial cells and resident leukocytes, which initiate the local inflammatory response [6]. These, along with oxidative events, disrupt epithelial tight junctions and compromise the blood–milk barrier [7,8]. When infection persists, chronic inflammation develops, leading to epithelial apoptosis, alveolar fibrosis and loss of secretory function [5,9]. Thus, the inflammatory process is necessary to contain pathogens but can be damaging when dysregulated or prolonged [4].

In southern Chile (characterized by humid, pasture-based dairy systems) *Staphylococcus aureus* and *Streptococcus uberis* are the predominant etiological agents of mastitis [10]. The persistence of *S. aureus* and *S. uberis* is linked to biofilm formation and intracellular invasion, which hinder antibiotic effectiveness and facilitate recurrence [11]. More recently, fungal mastitis, caused primarily by *Candida* species and opportunistic filamentous fungi, has emerged as a relevant issue, following prolonged antibiotic treatments [12]. *Pichia kudriavzevii* (formerly classified as *Candida krusei*) is among the most prevalent fungal pathogens associated with mastitis in southern Chile. In this region, 89.75% of mycotic mastitis cases were attributed to this yeast, accounting for 10.1% of all mastitis cases [13]. However, current therapeutic options are limited, as this species exhibits intrinsic resistance to fluconazole and reduced susceptibility to amphotericin B [14]. This highlights the need to identify effective alternative antifungal therapies.

The dry period constitutes a critical period for udder health, during which the mammary gland undergoes tissue remodeling that increases susceptibility to new intramammary infections. Traditionally, the blanket dry cow therapy (BDCT) approach, involving the administration of intramammary antibiotics to all quarters at drying-off, was considered the gold standard. While BDCT reduces infections, it substantially increases antibiotic use, residue risk and microbial selective pressure. In contrast, the selective dry cow therapy (SDCT) model, where only infected or high-risk quarters receive antibiotics and internal teat sealants are applied to healthy ones, has shown comparable or even superior outcomes when supported by diagnostic testing and proper management [15]. Nevertheless, its adoption in southern Chilean pasture-based systems faces challenges such as high prevalence of contagious pathogens, seasonal calving, herd dispersion and climatic conditions that favor pathogen persistence. These limitations underscore the need for complementary non-antibiotic strategies to ensure udder health and sustainable production [16].

Among the most promising alternatives are essential oils (EOs): complex mixtures of volatile secondary metabolites, mainly monoterpenes, sesquiterpenes and phenylpropanoids with antimicrobial, antifungal, anti-inflammatory and antioxidant activities [3,17]. Unlike conventional antibiotics, EOs act through multi-target mechanisms, including disruption of lipid bilayers, increased ion permeability, interference with quorum sensing and biofilm formation, and modulation of host immune responses [3,18]. These multimodal actions decrease the likelihood of resistance development and promote tissue diffusion due to their lipophilic nature [3]. In vitro studies from different regions demonstrate the potential activity of several EOs against major bovine mastitis pathogens such as *S. aureus* and *S. uberis*, with reported minimal inhibitory concentrations (MICs) typically ranging from 0.39 to 6.25 mg/mL under experimental conditions [19]. In addition, some studies have described synergistic interactions between EOs and β-lactams or macrolides, suggesting that EOs could enhance antibacterial efficacy and allow a reduction in antibiotic dosage [20]. However, these findings remain largely restricted to in vitro assays, involving a limited number of strains and not representative of region-specific pathogen profiles. In practice, the incorporation of EOs into veterinary mastitis-control protocols continues to be constrained by issues such as chemical variability and lack of standardization, formulation stability and bioavailability within the mammary gland, and the need to ensure milk safety [21].

Within this context, the Valdivian temperate rainforest of southern Chile constitutes a biogeographical reservoir of plant species with therapeutic potential. This ecosystem, characterized by high rainfall and evolutionary isolation, harbors a flora rich in bioactive metabolites. Among its native species, *Laureliopsis philippiana* (Tepa) and *Drimys winteri* (Canelo) stand out as ethnobotanically significant trees, traditionally used in Mapuche medicine for their analgesic, wound-healing and antimicrobial properties [22]. The EO of *L. philippiana* is rich in monoterpenes such as linalool and eucalyptol, compounds associated with antibacterial and antifungal activity. Recent studies have shown in vitro efficacy against *S. aureus*, *Klebsiella pneumoniae* and *Candida albicans*, together with low cytotoxicity in non-tumorigenic human mammary epithelial cells (MCF-10A) [20]. The EO of *D. winteri* (Winteraceae) contains drimane-type sesquiterpenes such as drimenol and polygodial, which exhibit antimicrobial, antifungal, antioxidant and anti-inflammatory activities [23,24]. These drimane compounds act primarily by disrupting fungal membranes and inducing lipid peroxidation, as well as interfering with mitochondrial ATP synthesis and redox enzyme function [24,25,26]. Most available evidence for *L. philippiana* and *D. winteri* EOs derives from studies on human or foodborne pathogens and phytopathogenic fungi, such as *S. aureus*, *Escherichia coli*, *K. pneumoniae*, *Candida* spp. and *Gaeumannomyces graminis*, or from non-mammary eukaryotic models [22,23,25,27,28]. To our knowledge, no work has systematically evaluated their activity against dairy-relevant mastitis pathogens such as *S. aureus*, *S. uberis* or *P. kudriavzevii*, which limits their integration into udder-health strategies tailored to southern Chilean pasture-based systems [3,29].

The growing limitations of antibiotic-based mastitis therapy further emphasize the need for alternative or complementary strategies within a One Health framework [30,31,32]. In this scenario, EOs emerge as attractive candidates due to their membrane-active, multitarget mechanisms and relatively low risk of resistance development [3,29]. Therefore, this study aimed to explore the therapeutic potential of *L. philippiana* and *D. winteri* EOs as non-antibiotic alternatives to improve udder health in dairy herds.

## 2. Materials and Methods

### 2.1. Collection, Identification, and Extraction

*L. philippiana* Mol. (Monimiaceae) and *D. winteri* J.R. et G. Forst leaves were collected at the beginning of the flowering season in October 2024 from Cutipay (39°49′52″ S 73°20′27″ W), Valdivia, Chile (the aerial parts). Botanist Sebastian Cani identified the plant, and a specimen was deposited in the Herbarium of the Faculty of Chemical and Pharmaceutical Sciences, University of Chile (No VALD 3560 and No VALD 3561). *L. philippiana* EO (LP_EO) and *D. winteri* EO (DW_EO) were obtained using the hydrodistillation (HD) technique in a Clevenger-type apparatus and stored in amber glass bottles. Residual air was displaced with gaseous nitrogen, and EOs were stored at −20 °C until further analysis, as described previously [33].

### 2.2. Essential Oil Analysis (GC-MS)

The extract was analyzed by gas chromatography coupled to mass spectrometry (GC-MS) to determine its chemical profile. Analyses were performed on a Shimadzu QP 2010 Plus instrument (Shimadzu, Kyoto, Japan) equipped with an Rtx-5MS fused-silica capillary column (30 m length, 0.25 mm internal diameter, 0.25 μm film thickness) operating under splitless injection conditions. Analytical conditions were as follows: helium served as the carrier gas at a constant flow rate of 1.0 mL/min, with an injection volume of 1.0 µL. Mass spectra (MS) were recorded in full-scan mode across a range of 35–500 *m*/*z* at a scan frequency of 1.56 scans/s. Electron impact ionization (EI+) was employed at 70 eV, with the ion source maintained at 280 °C. The oven temperature was initially set to 50 °C and held for 5 min, then increased at 8 °C/min to 280 °C, where it remained for an additional 15 min. Terpenes present in the EO were identified by comparing their retention indices (RI) with a homologous series of n-alkanes (C10–C30; Supelco^®^, Merck, Darmstadt, Germany) analyzed under identical experimental conditions. Standards, procured from Sigma-Aldrich^®^ (Merck, Darmstadt, Germany) confirmed to have 99% purity, were co-injected for validation. Compound identification relied on spectral matching against the NIST 05 and Wiley MS libraries (NIST/EPA/NIH Mass Spectral Library with Search Program, version 2.0 g; NIST data version 11, accessible online at http://www.nist.gov/srd/nist1a.cfm accessed on 25 November 2025) and comparison with previously reported MS data [22,34].

### 2.3. Antimicrobial Activity

#### 2.3.1. Isolation and Culture of Mastitis Pathogens

Nine dairy herds from Los Ríos Region, with a documented history of high bulk tank somatic cells counts (SCC), were selected for the study. All cows from these herds with an SCC > 200,000 in the most recent Dairy Herd Improvement (DHI) report were sampled. A sample from the affected mammary quarter was obtained from each high SCC cow and sent to the laboratory at 0–4 °C for bacteriological analysis. Eight isolates of *S. aureus*, nine of *S. uberis*, and eight of *P. kudriavzevii* were identified following National Mastitis Council (NMC) protocols [35] complemented with conventional PCR [36] for bacteria, and with API 20C AUX for yeasts, and frozen at −80 °C for further analysis. The reference strains *S. aureus* ATCC 25923, *S. aureus* ATCC 43300, and *S. uberis* ATCC 9927 were included in all bacteriological experiments. To confirm the performance and reproducibility of the yeast assay, *Candida parapsilosis* ATCC 22019 was included as a quality control strain in all antifungal experiments, but was not part of the comparative data analysis.

#### 2.3.2. Agar Disk Diffusion Assay

Clinical isolates and reference strains were previously thawed and then seeded on Mueller Hinton agar for *S. aureus*, blood agar for *S. uberis*, or Sabouraud dextrose agar for yeasts (*P. kudriavzevii* and *C*. *parapsilosis*) at 37 °C for 18 to 24 h. A microbial inoculum was prepared adjusting the density of the microbial suspension to a transmittance equivalent to 0.5 for bacteria or 1 for yeasts on the McFarland scale at 530 nm. Previously sterilized filter paper disks were immersed in the respective pure EOs, and then placed in each plate previously seeded with the bacteria or yeast and incubated aerobically at 37 °C for 18–24 h. Oxacillin was used as the reference antibacterial agent and amphotericin B as the reference antifungal agent. The diameter of the inhibition zone around each disk (diameter of the inhibition zone plus diameter of the disk) was measured in mm, indicating the antimicrobial activity of the respective oil [37].

#### 2.3.3. Microplate Assay

The microdilution method in 96-well plates was used to determine the MIC, following Clinical Laboratory Standards Institute guidelines [38,39]. Briefly, EOs were solubilized in dimethyl sulfoxide (DMSO), with a final DMSO concentration not exceeding 9.2%, and tested at twofold serial dilutions ranging from 0.03125 to 64 mg/mL. Dilutions were prepared in Mueller–Hinton broth (MHB; Oxoid, Hampshire, UK) for *S. aureus*, MHB supplemented with 6% fetal bovine serum for *S. uberis*, or MHB supplemented with RPMI 1640 (1:1 ratio) for yeasts. Fresh microbial suspensions, adjusted to a transmittance equivalent to 0.5 on the McFarland scale at 530 nm, were used to inoculate the microplates, which were then incubated at 37 °C for 18–24 h. Sterility control (broth only), growth control (broth + inoculum), antimicrobial control (Cefoxitin 4 mg/L for bacteria and amphotericin B 2 mg/mL for yeasts), and solvent control (broth + DMSO + inoculum) were included to rule out any antimicrobial effect of the solvent. After incubation, growth was evaluated by observing turbidity against a dark background and measuring absorbance at 600 nm in a plate reader (Varioskan, ThermoFisher Scientific, Waltham, MA, USA). The MIC was determined as the lowest concentration of EO that showed no visible growth or increase in absorbance, following CLSI guidelines. However, since it is not known whether the antifungal activity of EOs is fungistatic or fungicidal, both types of antimicrobial action were considered when performing the MIC analysis for yeasts. Assuming a fungistatic effect, the MIC was considered as the lowest concentration of EO whose optical density (OD) was ≤50% of the growth control well, and assuming a fungicidal effect, the MIC was considered as the lowest concentration of EO with OD ≤ 5% of the growth control [40]. All EOs concentrations and controls were tested in triplicate.

### 2.4. Statistical Analysis

All statistical analyses were conducted using R software version 4.4.2 (R Core Team, Vienna, Austria, 2024). Inhibition zone diameters from the disk diffusion test were compared between treatments using a Student’s *t*-test, after verifying normality with the Shapiro–Wilk test. MIC values were summarized by MIC_50_, MIC_90_, and range. Differences between oils or species were assessed using the Mann–Whitney U test when normality assumptions were not met.

## 3. Results

### 3.1. Composition

**Figure 1:**
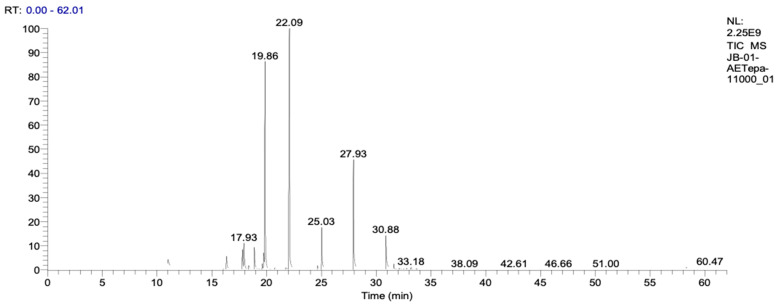
Chromatography profile of LP_EO. The peaks observed correspond to the major chemical compounds, mainly monoterpenes.

**Figure 2:**
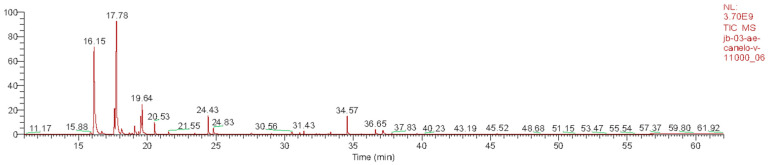
Chromatography profile of DW_EO. The peaks observed correspond to the major chemical compounds, mainly monoterpenes.

LP_EO and DW_EO were analyzed by GC–MS (Table 1, Figure 1; Table 2, Figure 2), revealing distinct profiles dominated by monoterpenes and sesquiterpenes. LP_EO was characterized by a high proportion of oxygenated and hydrocarbon monoterpenes, among which sabinene (13.95%), eucalyptol (12.05%) and terpinen-4-ol (7.03%) were the most abundant, compounds that have been associated with antimicrobial and anti-inflammatory properties. In contrast, DW_EO displayed a predominantly hydrocarbon monoterpenic profile, with β-pinene (35.70%) and α-pinene (25.30%) as the major constituents. These results are consistent with previous reports describing a similar hydrocarbon monoterpenic profile for DW_EO [28].

The identification of individual components was based on retention time (tr), experimental Kovats index (I.R.e, calculated relative to a C10–C30 n-alkane standard on an Rtx-5MS capillary column), literature Kovats index (I.R.t) and mass spectral matching (match factor > 75) against the NIST 14 library, complemented by co-elution with reference standards available in the laboratory. Area% corresponds to the relative area under the curve of each peak, indicating the proportion of each compound in the total sample; Id denotes compound identification and CAS refers to the Chemical Abstracts Service registry number. Colored quadrants in Table 1 and Table 2 highlight the major constituents in each EO.

### 3.2. Antibacterial Activity

A Welch’s *t*-test was conducted to compare the mean inhibition zone diameters between LP_EO and DW_EO within each bacterial species (Table 3). For *S. aureus*, the mean inhibition zone was significantly larger with DW_EO (9.73 mm) compared to LP_EO (7.24 mm) (*p*-value = 0.0104). For *S. uberis*, although the mean inhibition zone was also larger with DW_EO (8.45 mm) than with LP_EO (7.27 mm), this difference was not statistically significant (*p*-value = 0.07). When reference strains were evaluated separately, both EOs produced measurable inhibition zones against *S. aureus* and *S. uberis*. For *S. aureus* ATCC 25923, inhibition zones were 6 mm with LP_EO and 7 mm with DW_EO, while for the methicillin-resistant strain *S. aureus* ATCC 43300, LP_EO produced a 5 mm inhibition zone and DW_EO a 7 mm zone. For *S. uberis* ATCC 9927, both LP_EO and DW_EO generated identical inhibition zones of 6 mm.

The MICs of LP_EO and DW_EO were determined for clinical isolates and reference strains of *S. aureus* and *S. uberis* (Table 4). For *S. aureus*, DW_EO exhibited significantly lower MICs compared to LP_EO (*p*-value = 0.01). For DW_EO, the MIC_50_ was 16 mg/mL; meanwhile, for LP_EO, it was 64 mg/mL, indicating greater overall activity. Additionally, the MIC_90_ for both oils was 64 mg/mL. When reference strains were analyzed separately, DW_EO showed lower MIC values than LP_EO against both methicillin-resistant and methicillin-susceptible *S. aureus*. Specifically, DW_EO inhibited *S. aureus* ATCC 43300 (MRSA) at 8 mg/mL, compared to 64 mg/mL for LP_EO, while for *S. aureus* ATCC 25923 the MIC values were 2 mg/mL and 4 mg/mL for DW_EO and LP_EO, respectively. In contrast, *S. uberis* showed no statistically significant difference between oils (*p*-value = 0.09), suggesting comparable antibacterial activity. The MIC_50_ and MIC_90_ with DW_EO were 8 and 16 mg/mL, respectively, while for LP_EO, both were 16 mg/mL. The MIC range was also narrower for DW_EO (4.0–16.0 mg/mL) than for LP_EO (4.0–32.0 mg/mL). For the reference strain of *S. uberis*, both LP_EO and DW_EO exhibited identical MIC values (4 mg/mL), indicating similar inhibitory activity against this species under the tested conditions. These findings indicate that DW_EO has superior antimicrobial activity, particularly against *S. aureus*, and that both oils exhibit variable but measurable activity against Gram-positive mastitis pathogens.

### 3.3. Antifungal Activity

A Welch’s *t*-test was conducted to compare the mean inhibition zone diameters between LP_EO and DW_EO for *P. kudriavzevii* (Table 5). The mean inhibition zone was significantly larger with DW_EO (15.39 mm) compared to LP_EO (10.71 mm) (*p*-value = 0.03).

The MIC of LP_EO and DW_EO for clinical isolates of *P. kudriavzevii* was determined (Table 6). Assuming fungistatic activity, DW_EO showed a lower MIC_50_ (2 mg/mL) compared to LP_EO (4 mg/mL), indicating greater overall activity (*p*-value = 0.01). The MIC_90_ for DW_EO was 4 mg/mL, and for LP_EO, it was 8 mg/mL. On the other hand, assuming fungicidal activity, LP_EO showed MIC_50_ and MIC_90_ equal to 16 mg/mL, and DW_EO reached MIC_50_ and MIC_90_ of 8 mg/mL, suggesting more consistent efficacy in all isolates (*p*-value = 0.01). The MIC ranges varied from 0.5 to 16 mg/mL, depending on the EO analyzed. These findings indicate that DW_EO may have superior antifungal potency and that both oils exhibit variable but measurable activity against *P. kudriavzevii*, the causative agent of fungal mastitis.

## 4. Discussion

The search for alternatives and complements to antimicrobial therapy has become a central axis of modern mastitis control, particularly in pasture-based dairy systems where antimicrobial stewardship and residue-free production are increasingly demanded [3,29]. Within this context, plant EOs have attracted growing attention as multi-target agents with antibacterial and antifungal activity; however, most available data focus on a restricted set of cosmopolitan species and on human or foodborne pathogens rather than on bovine mastitis isolates.

Our study addresses this gap by providing a first in vitro characterization of the chemical composition and antimicrobial properties of EOs from two native Valdivian trees, *D. winteri* and *L. philippiana*, against *S. aureus*, *S. uberis* and *P. kudriavzevii* isolated from dairy cows in southern Chile. In the following sections, we interpret these findings in light of current knowledge on mastitis epidemiology, EO chemistry and antifungal resistance, and outline the potential role of these native EOs as candidates for further development within mastitis control programs.

### 4.1. Chemotaxonomic Basis for Activity and Mechanistic Considerations

The GC–MS profile provides a coherent basics to interpret the differences observed between both essencial oils. LP_EO was dominated by oxygenated and hydrocarbon monoterpenes, with sabinene, eucalyptol and terpinen-4-ol as major constituents, consistent with previous reports in agreement with previous reports for *L. philippiana* essential oil [41]. In contrast, DW_EO showed a predominantly hydrocarbon monoterpenic profile, largely driven by β-pinene and α-pinene, in agreement with earlier descriptions of *D. winteri* chemotypes and their biological activities [41,42]. In addition, drimanic sesquiterpenes described for *D. winteri* (e.g., drimenol and polygodial) have been associated with antimicrobial effects [42], supporting the view that DW_EO integrates both monoterpenic and sesquiterpenic contributors to bioactivity.

This compositional divergence is mechanistically relevant, as lipophilic monoterpenes readily partition into microbial membranes, a property commonly associated with increased membrane permeability, loss of membrane integrity, and disruption of essential cellular processes. This membrane-centered framework offers a direct explanation linking the pinene-rich profile of DW_EO to the consistently stronger antibacterial and antifungal activity observed in our assays, relative to LP_EO. Although oxygenated monoterpenes (e.g., terpinen-4-ol and eucalyptol) present in LP_EO may also contribute to antimicrobial effects, the overall chemical profile of DW_EO appears more consistent with the higher activity measured against the clinical isolates evaluated in the present study [22,43].

Among DW_EO constituents, α-pinene has been repeatedly associated with antifungal activity, and monoterpenes present in essential oils are widely recognized as key contributors to antifungal effects against a broad range of fungi [44]. In *Candida* spp., available evidence primarily supports membrane-associated mechanisms (including sterol-related alterations) and inhibition of biofilm formation, with additional data suggesting broader multitarget effects [45,46]. In this context, the combination of a high abundance of α/β-pinene with the reported presence of drimane-type sesquiterpenes in *D. winteri* [41,42] provides a plausible chemotaxonomic basis for the superior activity of DW_EO observed in the present study.

However, as the oils were assessed as whole mixtures, the present study does not allow the observed effects to be attributed to individual constituents, nor does it enable a quantitative assessment of potential synergistic or antagonistic interactions among compounds. Future research should therefore incorporate fractionation strategies, defined combination studies, and mechanism-oriented assays to identify the main bioactive contributors, clarify interaction effects, and strengthen the translation of these in vitro findings into practical applications for udder health.

### 4.2. Comparative Antibacterial Activity and Relevance for Mastitis Control

The antibacterial results confirm that both LP_EO and DW_EO are able to inhibit mastitis-associated *S. aureus* and *S. uberis* in vitro, but also reveal a consistent superiority of DW_EO. At comparable concentrations, DW_EO generated larger inhibition zones and lower MIC_50_/MIC_90_ values than LP_EO for both species, suggesting a stronger and more homogeneous effect across clinical isolates. This pattern is particularly relevant given that *S. aureus* and *S. uberis* are among the predominant etiological agents of mastitis in southern Chile and are characterized by robust biofilm formation and relevant antimicrobial resistance patterns [47], which frequently compromise conventional antibiotic therapy. Importantly, the methicillin-resistant type strain *S. aureus* ATCC 43300 (MRSA) was also included in the panel, and DW_EO showed measurable antimicrobial activity against this clinically relevant strain. However, biofilm production was not specifically characterized in the field isolates included in this study, and antibiofilm activity was therefore not directly assessed. Given that biofilm formation represents a major therapeutic challenge in chronic and recurrent mastitis, particularly for *S. aureus* [48], future studies should evaluate the activity of these EOs against biofilm-forming phenotypes, including effects on biofilm formation, maturation and disruption.

From an antimicrobial stewardship perspective, these in vitro data position DW_EO as a candidate for further preclinical evaluation as a topical prophylactic or adjunctive tool, rather than as a stand-alone replacement for antibiotics. This is compatible with current proposals that emphasize integrating alternative approaches into mastitis control programs to reduce antimicrobial reliance [29]. Two potential applications can be envisaged. First, incorporation of DW_EO into pre- and post-milking teat dips could help reduce bacterial load on teat skin and canal, potentially limiting colonization by *S. aureus* and *S. uberis* and early biofilm development. Second, EO-based formulations could be explored as complementary measures in SDCT programs, especially in pasture-based systems where diagnostic constraints hinder full implementation of culture-guided SDCT and environmental exposure is high [16].

However, translating these concepts into practice requires caution. We did not assess cytotoxicity in bovine mammary cells, effects on milk quality, or in vivo efficacy, and EO activity and toxicity depend on dose exposure time, and formulation [49]. Consequently, DW_EO should currently be regarded as a promising antibacterial candidate at the in vitro level, and further work is needed to develop standardized formulations, to assess stability and compatibility with existing teat dips or internal sealants, and to conduct controlled field trials.

### 4.3. Antifungal Activity Against P. kudriavzevii and Implications for Mycotic Mastitis

One of the most relevant contributions of this work is the demonstration of EO activity against *P. kudriavzevii*, a yeast increasingly implicated in bovine mycotic mastitis in southern Chile and recognized as a clinically relevant, azole-resistant species in the human and veterinary context [13]. Under our conditions, both EOs inhibited *P. kudriavzevii* growth in vitro, but DW_EO displayed a clearly higher effect, with larger inhibition zones and lower MIC values than LP_EO. Notably, DW_EO achieved fungicidal endpoints (OD ≤ 5%) at the tested concentrations, whereas LP_EO mainly exhibited fungistatic effects (OD ≤ 50%).

These findings are consistent with evidence that EOs can exert antifungal activity through multi-target actions affecting membrane integrity and associated cellular processes, among other pathways [17,49]. Prior work also indicates that DW_EO and drimanic sesquiterpenes from *D. winteri* can display strong antimicrobial activity against resistant bacteria and yeast [41,42], supporting the idea that DW_EO contains multiple active scaffolds.

From a clinical standpoint, this dual antibacterial–antifungal profile is particularly valuable for pasture-based dairy systems, where fungal mastitis may emerge following prolonged antibiotic therapy and where treatment options are limited by resistance patterns and residue concerns. Overall, our in vitro data support DW_EO as a high-potential antifungal candidate for further preclinical development, including topical or intramammary delivery concepts. Nonetheless, this will require targeted toxicological evaluation of udder skin and mammary tissue, optimization of formulation and delivery, plus well designed in vivo and on farm studies to establish efficacy and safety.

### 4.4. Implications for Sustainable Udder Health

Beyond the direct benefits of reducing antimicrobial use, the phytotherapeutic approach contributes to the broader goals of sustainable udder health [29,30,31]. The dual antibacterial–antifungal profile of DW_EO is critical for antimicrobial stewardship, mitigating AMR development [3,16]. The use of EOs is often cited as a tool in alternative strategies due to their multi-target mechanisms, which decreases the likelihood of resistance development compared to single-target conventional drugs [17,49].

Furthermore, the localized, pasture-based dairy systems of southern Chile can benefit economically from the development of a regional value chain based on native species, potentially reducing reliance on expensive imported pharmaceuticals and promoting the economic viability and resilience of local farmers [16]. Essential oils, as natural products, are generally more environmentally compatible and biodegradable than many synthetic antibiotic residues, reducing the environmental load. Thus, the exploration of native EOs supports a system-level solution that enhances economic, environmental, and animal health viability within a comprehensive One Health framework in local dairy production [3,29]. However, caution is required, and future work should include life cycle assessment comparing the environmental footprint of EOs production with conventional antibiotic manufacturing, systems level economic analyses of farmer viability, and address risks of plant overexploitation by assessing collection quotas and cultivation potential, to support sustainability claims beyond reduced antimicrobial use.

## 5. Conclusions

This study provides the first integrative in vitro assessment of the chemical composition and antimicrobial potential of *L. philippiana* and *D. winteri* EOs against key bovine mastitis pathogens. The two oils exhibited distinct chemotypes, with *D. winteri* characterized by a pinene-rich profile and *L. philippiana* by higher levels of oxygenated monoterpenes. These differences were reflected in their biological activity: while both oils inhibited *S. aureus*, *S. uberis*, and *P. kudriavzevii*, *D. winteri* consistently showed superior antibacterial activity and was the only oil to demonstrate clear fungistatic and fungicidal effects against the azole-resistant yeast *P. kudriavzevii.*

The dual antibacterial–antifungal profile of *D. winteri* positions this native species as a promising phytotherapeutic candidate at the preclinical stage. However, these findings remain confined to controlled in vitro conditions. Future studies to advance DW_EO toward practical implementation must include complementary mechanistic studies, cytotoxicity and safety assessments in bovine mammary tissues, optimization of delivery systems, and evaluation of efficacy in vivo and under field conditions.

Overall, our results highlight the potential of Chilean Valdivian flora as a source of locally adapted therapeutic leads and provide a scientific foundation for developing plant-based adjuncts that support antimicrobial stewardship and sustainable udder-health management in pasture-based dairy systems.

## Figures and Tables

**Table 1:** Results of GC–MS analysis of *Laureliopsis philippiana* essential oil (LP_EO).

Compound Name	CAS	RT (min)	KI exp	Area %
beta-Thujene	28634-89-1	16.1	929.6	0.71
α-Pinene	80-56-8	16.36	937.1	4.38
Camphene	79-92-5	16.93	953.1	0.57
**Sabinene**	**3387-41-5**	**17.81**	**976.7**	**13.95**
β-Pinene	18172-67-3	17.96	980.7	5.72
β-Myrcene	123-35-3	18.36	990.9	1.25
α-Phellandrene	99-83-2	18.92	1006.5	0.26
α-Terpinolene	99-86-5	19.32	1019.2	2.36
p-Cymene	99-87-6	19.6	1028	2.51
D-Limonene	5989-27-5	19.74	1032.3	2.7
**Eucalyptol**	**470-82-6**	**19.86**	**1036**	**12.05**
β-Ocimene	13877-91-3	20.31	1049.7	2.85
γ-Terpinene	99-85-4	20.74	1062.4	4.09
α-Terpinolene	586-62-9	21.75	1091.4	0.95
Linalool	78-70-6	22.04	1099.4	0.96
Butanoic acid, 2-methyl-, 3-methyl-3-butenyl ester	84254-81-9	22.4	1111.7	2.18
Butanoic acid, 3-methyl-, 3-methyl-3-butenyl ester	54410-94-5	22.5	1115.1	1.02
Neo-allo-ocimene	7216-56-0	22.99	1131.5	0.26
**Terpinen-4-ol**	**562-74-3**	**24.64**	**1184.4**	**7.03**
L-α-Terpineol	10482-56-1	25.06	1197.3	0.28
δ-Elemene	20307-84-0	29.24	1345.6	1.14
α-Cubebene	17699-14-8	29.58	1358.2	0.64
Copaene	3856-25-5	30.38	1387.2	0.67
β-Elemene	515-13-9	30.73	1399.6	1.5
cis-α-Bergamotene	18252-46-5	31.3	1422.8	0.15
Caryophyllene	87-44-5	31.62	1435.7	1.2
trans-α-Bergamotene	13474-59-4	31.82	1443.7	2.91
Guaia-6,9-diene	36577-33-0	32.12	1455.5	3.44
Cadina-3,5-diene	267665-20-3	32.38	1465.7	0.41
Humulene	6753-98-6	32.5	1470.4	1.09
Germacrene D	23986-74-5	33.17	1496.2	2.23
Bicyclogermacrene	24703-35-3	33.56	1512.4	1.26
Calamenene	483-77-2	34.15	1537.3	3.24
α-Calacorene	21391-99-1	34.69	1559.8	0.22
trans-Nerolidol	40716-66-3	34.87	1567.2	1.68
Furopelargone A	1143-45-9	35.66	1599.2	4.65
Caryophyllene oxide	1139-30-6	35.77	1604.1	0.59
Humulene epoxide II	19888-34-7	36.38	1631.5	0.44
Cubenol	21284-22-0	36.72	1646.6	0.4
Eudesmol	473-15-4	37.36	1674.6	0.58
Kaur-16-ene	562-28-7	45.67	2083.7	0.27

Bold quadrants highlight the major constituents in LP_EO: sabinene (13.95%), eucalyptol (12.05%) and terpinen-4-ol (7.03%). CAS: Chemical abstract service number. RT: Retention indices. KI exp: experimental Kovats indices.

**Table 2:** Results of GC–MS analysis of *Drimys winteri* essential oil (DW_EO).

Compound Name	CAS	RT	KI exp	Area %
α-Thujene	5-2-2867	15.88	923.2	0.5
**α-Pinene**	**80-56-8**	**16.15**	**931.1**	**25.3**
Camphene	79-92-5	16.7	946.7	0.8
Sabinene	3387-41-5	17.61	971.5	5.6
**β-Pinene**	**18172-67-3**	**17.77**	**975.7**	**35.7**
β-Myrcene	123-35-3	18.14	985.3	1.2
α-Phellandrene	99-83-2	18.7	999.5	0.5
3-Carene	13466-78-9	18.89	1005.5	0.3
α-Terpinene	99-86-5	19.1	1012.2	1.9
p-Cymene	99-87-6	19.4	1021.7	0.5
D-Limonene	5989-27-5	19.53	1025.8	5
Eucalyptol	470-82-6	19.64	1029.2	5.9
3-Carene	13466-78-9	20.22	1047	n.d
γ-Terpinene	99-85-4	20.53	1056.2	2.5
α-Terpinolene	586-62-9	21.55	1085.8	0.7
Neo-allo-ocimene	7216-56-0	22.8	1125.2	n.d
Camphor	76-22-2	23.47	1147.3	0.2
Terpinen-4-ol	562-74-3	24.43	1177.8	3.6
α-Terpineol	98-55-5	24.83	1190.2	1.2
Bornyl acetate	5655-61-8	27.6	1285.5	0.2
Safrole	94-59-7	27.73	1289.8	n.d
γ-Elemene	29873-99-2	29.07	1339.2	0.1
α-Cubebene	17699-14-8	29.39	1351.1	n.d
Copaene	3856-25-5	30.19	1380.4	n.d
β-Elemene	515-13-9	30.56	1393.6	0.4
α-Gurjunene	489-40-7	31.14	1416.3	0.3
Caryophyllene	87-44-5	31.43	1428.1	0.6
γ-Muurolene	30021-74-0	31.64	1436.5	n.d
Guaia-6,9-diene	36577-33-0	31.94	1448.4	n.d
Isogermacrene D	317819-80-0	32.04	1452.4	n.d
Cadina-3,5-diene	267665-20-3	32.19	1458.3	n.d
Humulene	6753-98-6	32.32	1463.4	n.d
β-Himachalene	1461-03-6	32.72	1478.9	n.d
α-Curcumene	644-30-4	32.8	1482	n.d
Germacrene D	23986-74-5	33	1489.7	n.d
Bicyclogermacrene	24703-35-3	33.38	1504.7	0.5
β-Curcumene	28976-67-2	33.52	1510.7	n.d
γ-Cadinene	39029-41-9	33.77	1521.3	n.d
Myristicin	607-91-0	33.86	1525.1	n.d
δ-Cadinene	483-76-1	33.92	1527.7	n.d
Cadina-1(2),4-diene	16728-99-7	34.2	1539.4	n.d
Italicene ether	104188-25-2	34.31	1544	n.d
Elemol	639-99-6	34.58	1555.2	3.3
trans-Nerolidol	40716-66-3	34.69	1559.8	n.d
Spathulenol	6750-60-3	35.42	1589.5	n.d
Furopelargone A	1143-45-9	35.48	1592	n.d
Caryophyllene oxide	1139-30-6	35.59	1596.4	n.d
Globulol	51371-47-2	35.61	1597.2	0.1
Viridiflorol	552-02-3	35.8	1605.4	n.d
Di-epi-1,10-cubenol	73365-77-2	36.54	1638.6	n.d
γ-Eudesmol	1209-71-8	36.65	1643.5	1
β-Acorenol	28400-11-5	36.65	1643.5	n.d
τ-Cadinol	11-1-5937	36.8	1650.1	n.d
Eudesmol	473-15-4	37.14	1665	0.7
α-Cadinol	481-34-5	37.14	1665	n.d
α-Eudesmol	473-16-5	37.2	1667.6	0.7
Kaur-16-ene	562-28-7	45.52	2075.8	0.2

Bold quadrants highlight the major constituents in DW_EO: α-pinene (25.3%) and β-pinene (35.7%). CAS: Chemical abstract service number. RT: Retention indices. KI exp: experimental Kovats indices. n.d: not determined.

**Table 3:** Comparison of inhibition zones (mm) between LP_EO and DW_EO for *S. aureus* and *S. uberis*.

Species	EO	Mean	Median	SD	Min	Max	Number of Isolates
*Staphylococcus aureus*	DW_EO	9.73 *	10.2	2.15	7	13.6	10
*Staphylococcus aureus*	LP_EO	7.24 *	7	1.69	5	10	10
*Streptococcus uberis*	DW_EO	8.70	8.75	2.02	6	12	10
*Streptococcus uberis*	LP_EO	7.30	7.00	0.95	6	9	10

LP_EO: *Laureliopsis philippiana* essential oil. DW_EO: *Drimys winteri* essential oil. * Statistical significance *p*-value < 0.05.

**Table 4:** Comparison of MIC between LP_EO and DW_EO for *S. aureus* and *S. uberis*.

Species	EO	MIC_50_ (mg/mL)	MIC_90_ (mg/mL)	Range (mg/mL)
*Staphylococcus aureus*	DW_EO	16.0	64.0	2.0–64.0
*Staphylococcus aureus*	LP_EO	64.0	64.0	4.0–64.0
*Streptococcus uberis*	DW_EO	8.0	16.0	4.0–16.0
*Streptococcus uberis*	LP_EO	16.0	16.0	4.0–32.0

LP_EO: *Laureliopsis philippiana* essential oil. DW_EO: *Drimys winteri* essential oil. MIC: Minimal Inhibitory Concentration.

**Table 5:** Comparison of inhibition zones (mm) between LP_EO and DW_EO for *P. kudriavzevii*.

Species	EO	Mean	Median	SD	Min	Max	Number of Isolates
*Pichia kudriavzevii*	DW_EO	15.39 *	15.95	3.84	10.7	20.15	8
*Pichia kudriavzevii*	LP_EO	10.71 *	10.57	1.50	8.87	12.61	8

LP_EO: *Laureliopsis philippiana* essential oil. DW_EO: *Drimys winteri* essential oil. * Statistical significance *p*-value < 0.05.

**Table 6:** Comparison of the fungicidal (OD ≤ 5%) or fungistatic (OD ≤ 50%) activity between LP_EO and DW_EO for *P. kudriavzevii*.

Species	EO	MIC_50_ (mg/mL)	MIC_90_ (mg/mL)	Range (mg/mL)
*Pichia kudriavzevii (OD50%)*	LP_EO	4.0	8.0	4.0–8.0
*Pichia kudriavzevii (OD50%)*	DW_EO	2.0	4.0	0.5–4.0
*Pichia kudriavzevii (OD5%)*	LP_EO	16.0	16.0	8.0–16.0
*Pichia kudriavzevii (OD5%)*	DW_EO	8.0	8.0	4.0–8.0

LP_EO: *Laureliopsis philippiana* essential oil. DW_EO: *Drimys winteri* essential oil. MIC: Minimal Inhibitory Concentration.

## Data Availability

The raw data supporting the conclusions of this article will be made available by the authors on request.

## Abbreviations

The following abbreviations are used in this manuscript:

AMR	Antimicrobial resistance
EO	Essential oil
GC-MS	Gas Chromatography coupled with Mass Spectrometry
MIC	Minimum inhibitory concentration
BDCT	Blanket Dry Cow Therapy
SDCT	Selective Dry Cow Therapy
LP_EO	*L. philippiana* EO
DW_EO	*D. winteri* EO
HD	Hydrodistillation
MS	Mass spectra
RI	Retention indices
SCC	Somatic cells count
DHI	Dairy Herd Improvement
CLSI	Clinical Laboratory Standards Institute guidelines
EI+	Electron impact ionization
DMSO	Dimethyl sulfoxide
MHB	Mueller–Hinton broth
CAS	Chemical Abstract service number
KI	Kovats indices
OD	Optical density
